# Wood smoke exposure affects lung aging, quality of life, and all-cause mortality in New Mexican smokers

**DOI:** 10.1186/s12931-022-02162-y

**Published:** 2022-09-08

**Authors:** Shuguang Leng, Maria A. Picchi, Paula M. Meek, Menghui Jiang, Samuel H. Bayliss, Ting Zhai, Ruslan I. Bayliyev, Yohannes Tesfaigzi, Matthew J. Campen, Huining Kang, Yiliang Zhu, Qing Lan, Akshay Sood, Steven A. Belinsky

**Affiliations:** 1 Department of Internal Medicine, School of Medicine, University of New Mexico, Albuquerque, NM 87131 USA; 2 Cancer Control and Population Sciences, University of New Mexico Comprehensive Cancer Center, Albuquerque, NM 87131 USA; 3 Lung Cancer Program, Lovelace Biomedical Research Institute, Albuquerque, NM 87108 USA; 4 College of Nursing, University of Utah, Salt Lake City, UT 84112 USA; 5 Department of Environmental Health, Harvard T.H. Chan School of Public Health, Boston, MA 02115 USA; 6 Pulmonary and Critical Care Medicine Division, Department of Medicine, Brigham and Women’s Hospital, Harvard Medical School, Boston, MA 01255 USA; 7 College of Pharmacy, University of New Mexico, Albuquerque, NM 87131 USA; 8 Division of Cancer Epidemiology and Genetics, National Cancer Institute, National Institutes of Health, Rockville, MD USA

**Keywords:** Wood smoke, Lung function decline, Health related quality-of-life, Lung cancer, Mortality

## Abstract

**Background:**

The role of wood smoke (WS) exposure in the etiology of chronic obstructive pulmonary disease (COPD), lung cancer (LC), and mortality remains elusive in adults from countries with low ambient levels of combustion-emitted particulate matter. This study aims to delineate the impact of WS exposure on lung health and mortality in adults age 40 and older who ever smoked.

**Methods:**

We assessed health impact of self-reported “ever WS exposure for over a year” in the Lovelace Smokers Cohort using both objective measures (i.e., lung function decline, LC incidence, and deaths) and two health related quality-of-life questionnaires (i.e., lung disease-specific St. George's Respiratory Questionnaire [SGRQ] and the generic 36-item short-form health survey).

**Results:**

Compared to subjects without WS exposure, subjects with WS exposure had a more rapid decline of FEV1 (− 4.3 ml/s, P = 0.025) and FEV1/FVC ratio (− 0.093%, P = 0.015), but not of FVC (− 2.4 ml, P = 0.30). Age modified the impacts of WS exposure on lung function decline. WS exposure impaired all health domains with the increase in SGRQ scores exceeding the minimal clinically important difference. WS exposure increased hazard for incidence of LC and death of all-cause, cardiopulmonary diseases, and cancers by > 50% and shortened the lifespan by 3.5 year. We found no evidence for differential misclassification or confounding from socioeconomic status for the health effects of WS exposure.

**Conclusions:**

We identified epidemiological evidence supporting WS exposure as an independent etiological factor for the development of COPD through accelerating lung function decline in an obstructive pattern. Time-to-event analyses of LC incidence and cancer-specific mortality provide human evidence supporting the carcinogenicity of WS exposure.

**Supplementary Information:**

The online version contains supplementary material available at 10.1186/s12931-022-02162-y.

## Background

Wood smoke (WS), as a major contributor to ambient and indoor combustion emitted particulate matter (PM), has emerged as a critical public health issue in the USA and other high-income countries. As a result of global climate change, wildland fire events have steadily increased over the past 15 years in the USA and other areas of the world and created catastrophically high levels of fine PM (PM2.5) for weeks to months in the affected areas, with toxic smoke travelling to areas hundreds miles away [1]. In addition, WS emitted by wood stoves in millions of USA homes dramatically compromises indoor air quality and is also a major contributor (> 50%) to ambient PM pollution in many urban and rural communities during winter [2–5]. Studies of rural, wood stove heated homes in the USA found 30% of households having daily PM2.5 levels beyond the USA Environmental Protection Agency (EPA) limit (35 μg/m^3^) with peak levels resembling those seen in low-income countries [6, 7]. Recreational wood burning and prescribed fires also generate WS exposure, although with much less impact on the air quality.

Epidemiologic studies have linked WS exposure with increased prevalence of respiratory infections, symptoms, and diseases, cancer incidence, and deaths in children and adults in the settings of household heating and cooking, street cooking, fish or meat smoking, or charcoal production in low-income countries where PM2.5 levels from wood burning in those settings could be very high (> 500 µg/m^3^) [8, 9]. However, WS exposure is typically much lower in the US, which raises uncertainty for the direct extrapolation of the health findings from those high exposure scenarios [10]. Associations with respiratory symptoms or lung function decrement among children living in homes heated by a wood stove were identified in several USA communities [11, 12]. Sustained effects on lung function indicative of airway obstruction were also observed for 2 years following a 45-day exposure to wildfire smoke in Seeley Lake, Montana with daily average PM_2.5_ levels at 220.9 µg/m^3^ [1]. Our cross-sectional analyses using the Lovelace Smokers Cohort (LSC) identified associations between “self-reported ever WS exposure for over a year” and a lower percent predicted FEV1 and a higher prevalence of airflow obstruction and chronic mucous hypersecretion (CMH) [13]. However, the impact of WS exposure on lung function decline and its sequelae (e.g., lung cancer [LC] incidence, and disease mortality) in middle-aged and older adults has not been adequately addressed [14–18], particularly in high-income countries.

Disease-specific or generic health-related quality-of-life (HRQoL) questionnaires provide a holistic and quantitative approach assessing the multi-dimensional impacts (e.g., physical, psychological, and social aspects) of diseases or environmental exposures on health in clinical and research settings [19–21]. The St. George's Respiratory Questionnaire (SGRQ) is a lung disease-specific instrument whereas the 36-item short-form health survey (SF-36) is a generic HRQoL tool [22]. One study conducted in rural Bolivia found that ventilation remediation of biomass-burning cooking stoves greatly improved the SGRQ scores, supporting the responsiveness of SGRQ scores to PM driven indoor air quality [23].

Retrospective assessment of total WS exposure from all major sources (e.g., indoor and outdoor) in community-based studies is very challenging. Indoor WS exposure history was often assessed using self-report qualitative or semi-quantitative questionnaires developed by individual studies which are usually lack of standardization [24, 25]. Occupational WS exposure for firefighters, trappers/hunters, rangers, and cooks using wood fired appliances was barely considered in most if not all job exposure matrixes [26, 27]. Health effect studies of wildfire exposure mainly use exposure versus non-exposure comparison approach to identify health differences at aggregate level post exposure [1]. A single question “Have you ever been exposed to WS for 12 months or longer” was implemented in the Lovelace Smokers cohort at baseline to presumably provide a qualitative assessment (yes or no) of cumulative WS exposure for at least 1 year from any exposure sources [13]. With this binary exposure assessment, we identified a strong association between WS exposure and a lower percent predicted FEV1 and a higher prevalence of airflow obstruction and CMH, supporting the validity of this question to some degree [13]. In this study, we further assessed the impact of WS exposure in the LSC using both objective measures (i.e., lung function decline, LC incidence, and death) and two self-assessed HRQoLs (e.g., SGRQ and SF-36). The goal was to find epidemiological evidence supporting (1) the role of WS exposure on the development of chronic obstructive pulmonary disease (COPD) and (2) carcinogenicity of WS exposure in humans.

## Methods

### Study population

The LSC was established in 2001 to study sputum and blood biomarkers for LC risk assessment and COPD development in current and former smokers enrolled from the greater Albuquerque area of New Mexico. Study design and inclusion/exclusion criteria were described elsewhere [28, 29]. In brief, subjects were included in the study if they were 40–75 years of age, former or current smokers with at least 10 pack-years of smoking history, free of prior LC history, and able to understand English. At study entry, cohort members completed a battery of questionnaires including demographics, smoking and medical history, Harvard food frequency questionnaire, SF-36, and SGRQ, underwent pre- and post- bronchodilator spirometry testing adhering to the 1994 American Thoracic Society guidelines [30], and provided blood (lymphocytes, plasma) and induction sputum samples. Cohort members returned every 18 months to update smoking status, general health status and respiratory symptoms, undergo spirometry, and provide biological samples. Active enrollment and on-site follow-up ended in the summer of 2017. Spirometry data were collected from 2511 unique subjects for a total of 11,328 person-visits. Since then, living cohort members are contacted annually by phone calls or mail to collect data for LC incidence or death. This study was approved by the Western Institutional Review Board and all participants signed consent forms. Association analyses in this study were conducted in 2372 LSC subjects with at least one post-bronchodilator spirometry and no missing data for WS exposure and covariates. One hundred and thirty nine LSC subjects were excluded from this study mainly due to missing WS exposure (n = 126) and lack of at least one complete spirometry test (n = 9). Compared with subjects (n = 2372) included in this study, excluded subjects had similar age, BMI, smoking history, and prevalence of airway obstruction (defined as FEV1/FVC ratio ≤ 70% [31]) and CMH (defined as self-reported cough productive of phlegm for at least 3 months per year for at least 2 consecutive years [i.e., the standard definition of chronic bronchitis] [13]) at baseline.

### Definition of wood smoke exposure

WS exposure was self-reported in response to a question “Have you been exposed to wood smoke for 12 months or longer” as part of the general health survey at study entry.

### Health-related quality-of-life

Health-related quality-of-life (HRQoL) was assessed using the generic health SF-36 questionnaire and the lung disease-specific SGRQ with the recall period of past 4 weeks [32, 33]. The SF-36 encompasses eight domains including physical functioning, role physical, role emotional, social functioning, mental health, vitality, general health perceptions, and bodily pain. The SF-36 scores range from 0 to 100, with higher scores indicating better HRQoL [32]. The SGRQ total score and its activity, symptom, and impact domain subscores range from 0 to 100, with higher score indicating a worse HRQoL [34]. A minimal clinically important difference in SGRQ total score and domain subscores is 4 [35]. SGRQ was collected for all cohort members at baseline and then predominantly for those with FEV1/FVC ratio < 75% at follow-up visits, whereas SF-36 was only collected at baseline visit.

### Mortality and lung cancer incidence data

Two National Death Index (NDI) searches were completed in 2014 and mid 2020 and in total identified 380 deaths out of the 2372 subjects (Table 1). Primary causes of death were coded using International Classification of Diseases-10 (ICD-10). Incident lung cancer (n = 72) was identified through NDI searches, obituary data, or by self-report from study subjects or their next of kin. Pathology reports were collected to confirm diagnosis, cancer histology and stage.

**Table 1:** Characteristics of LSC subjects with and without ever WS exposure for over a year

Variable	WS exposure for over a year		P
	Ever	Never	
N	684	1688	
Baseline variables			
Age (year, mean ± SD)	55.3 ± 9.0	56.7 ± 9.5	0.0013*
Male sex (n, %)	188 (27.5)	438 (26.0)	0.44†
Ethnicity (n, %)			< 0.0001†
NHW	457 (66.8)	1286 (76.2)	
Hispanic	165 (24.1)	271 (16.1)	
Other ethnicities	62 (9.1)	131 (7.8)	
Current smoker (n, %)	425 (62.1)	957 (56.7)	0.015†
Packyears (median, IQR)	35.5 (26.8–48.5)	34.5 (25.5–48.5)	0.35‡
Time since quit (year, median, IQR)	6.3 (2.2–15.4)	9.4 (3.5–18.3)	0.0026‡
College education (n, %)††	450 (66.0)	1190 (70.7)	0.024†
Annual income ≥ 30 K (n, %)‡‡	248 (44.6)	684 (52.2)	0.0034†
FEV1 (L/s, mean ± SD)	2.54 ± 0.77	2.59 ± 0.76	0.21*
FVC (L, mean ± SD)	3.51 ± 0.94	3.53 ± 0.95	0.73*
FEV1/FVC ratio (%, mean ± SD)	72.3 ± 11.3	73.4 ± 10.2	0.024*
Airway obstruction (n, %)	215 (31.4)	442 (26.2)	0.0097†
CMH (n, %)	222 (32.4)	398 (23.5)	< 0.0001†
Sputum MI (median, IQR)§	2 (1–4)	2 (1–4)	0.90‡
Charlson comorbidity score ≥ 1 (n, %)	412 (60.2)	819 (48.5)	< 0.0001†
HRQoL: SF-36 (mean ± SD, % with score 100)			
Physical functioning	71.0 ± 27.3, 15.5||	78.1 ± 24.1, 22.1	< 0.0001*
Role physical	67.8 ± 40.5, 55.9||	79.1 ± 35.0, 69.1	< 0.0001*
Bodily pain	61.6 ± 26.2, 17.4||	68.8 ± 24.8, 24.9	< 0.0001*
Role emotional	69.7 ± 41.7, 61.7||	77.1 ± 38.5, 71.3	< 0.0001*
Social functioning	75.5 ± 27.2, 41.8||	82.2 ± 24.4, 52.7	< 0.0001*
Mental health	70.1 ± 20.2, 3.2**	74.9 ± 19.4, 5.3	< 0.0001*
Vitality	51.1 ± 23.8, 1.3	57.5 ± 21.9, 1.9	< 0.0001*
General health perceptions	61.8 ± 22.6, 3.7	68.7 ± 21.1, 4.9	< 0.0001*
HRQoL: SGRQ (mean ± SD, % with score 0)			
Symptom	39.1 ± 24.9, 5.3||	28.1 ± 22.2, 11.9	< 0.0001*
Activity	38.1 ± 25.9, 8.5||	29.2 ± 24.5, 16.1	< 0.0001*
Impact	16.2 ± 16.2, 18.7||	10.3 ± 13.4, 35.6	< 0.0001*
Total	27.8 ± 19.54, 1.0||	19.6 ± 17.0, 4.3	< 0.0001*
Longitudinal data			
# Spirometry (median, IQR)	3 (1.5–6)	4 (2–7)	0.0008‡
Duration in cohort (year, median, IQR)	3.6 (0.5–8.5)	4.6 (1.4–9.6)	0.0013‡
# SGRQ (median, IQR)	2 (1–5)	2 (1–6)	0.12‡
# Death by 2020 (n, %)	108 (15.8)	272 (16.1)	0.85†
Age at death (year, mean ± SD)	69.3 ± 10.3	72.8 ± 10.2	0.0029*
Primary cause of death (n, %)			0.90†
Cardio pulmonary diseases	41 (38.0)	114 (41.9)	
Cancers	35 (32.4)	84 (30.9)	
Other causes	32 (29.6)	74 (27.2)	
Lung cancer incidence (n, %)	22 (3.2)	50 (3.0)	0.74†
Age at LC diagnosis (year, mean ± SD)	68.6 ± 8.2	69.9 ± 8.2	0.56*

*CMH* chronic mucous hypersecretion, *HRQoL* health related quality of life, *IRQ* inter quartile range, *NHW* non-Hispanic white, *SD* standard deviation, *SGRQ* St. George’s Respiratory questionnaire, *MI* methylation index, *WS* woodsmoke

*Student t test. Age at death was missing for one subject with ever WS exposure

†Chi-square test

‡Wilcoxon rank sum test

§Sputum MI is available in 521 subjects with ever WS exposure for over a year and 1280 subjects without

||Chi-square test, P < 0.0001, compared to never WS exposure

**Chi-square test, P = 0.028, compared to never WS exposure

††Missing education for 2 subjects with ever and 5 subjects with never WS exposure

‡‡Missing income for 128 subjects with ever and 373 subjects with never WS exposure

### Gene promoter methylation in sputum

Promoter methylation of a 12-gene panel originally optimized for LC risk stratification in smokers by our group was measured in eligible sputum samples using nested methylation-specific PCR [36–38]. These 12 genes (Additional file 1: Table S1) were selected based on their strong cancer relevance, diversity of function, and specificity of methylation in lung epithelial cells. Methylation status for each individual gene was scored as 0 (unmethylated) or 1 (methylated). A composite methylation index (MI) was created that summed the number of genes methylated.

### Statistical analyses

Linear mixed effects (LME) models with a subject-specific random intercept and slope were used to assess whether WS exposure was associated with a more rapid decline of lung function (FEV1, FVC, and FEV1/FVC ratio) [38, 39]. An interaction term between WS exposure and time in cohort (TIC) and their main effects were included in the LME models. Fixed effects for baseline age, smoking status (current versus former), packyears of smoking, body mass index (BMI), and height, sex, and ethnicity (Hispanic and Others with NHW as the reference) were included in the LME models. A significant estimate for the interaction term indicates the slope of lung function change over time varies by WS exposure with the slope difference estimated by the coefficient of the interaction term. A significant main effect of WS exposure quantifies the difference in baseline lung function (i.e., intercept) between subjects with and without WS exposure. We further extended the LME models by including additional interaction terms between candidate variables (e.g., baseline current smoking, airway obstruction, and CMH) and TIC. Those analyses can further reveal whether baseline subject characteristics such as smoking status, airway obstruction, and CMH might further moderate or mediate the WS exposure and TIC interaction. Similar to the analyses of lung function, LME models with an additional adjustment for education levels (some college or above versus high school or lower) were used to assess whether WS exposure affects overall levels of SGRQ scores over time. Because SF-36 score was only available at baseline, we used linear models to assess the impact of WS exposure with adjustment for baseline age, smoking status, packyears, BMI, and education levels, sex, and ethnicity. Alternative models added age unadjusted Charlson comorbidity score (≥ 1 versus 0), airway obstruction, and CMH at baseline to assess the independent component of effects for WS exposure on SGRQ and SF-36 scores. Kaplan–Meier curve and Cox proportional hazards model assessed the impact of WS exposure on LC incidence, all-cause mortality, and cause-specific mortality. When assessing disease-specific mortality, deaths due to other or unknown causes were treated as competing risk. Baseline age, smoking status, packyears, BMI, and income, education, sex, and ethnicity were adjusted in the Cox model. Mediation analysis with permutation-based statistics was used to quantify the impact of subjective and objective health measurements on the associations between WS exposure and all-cause mortality. Addition analyses were conducted to address how likely the associations seen was due to differential misclassification or confounding by social-economic status and whether excluding cohort members with lung cancer incidence or who died shortly (< 4.86 year) after enrollment affect the associations seen in the entire cohort. All statistical analyses were conducted using SAS 9.4.

## Results

### Demographics of cohort members with and without WS exposure

Approximately 29% of LSC subjects self-reported to be “ever exposed to WS for over a year” at baseline interview (Table 1). Those subjects were slightly younger and more likely to be Hispanic and current smokers, and had shorter abstinence period if quit smoking. Subjects with WS exposure had lower education and annual income reported at enrollment. Subjects with WS exposure also had shorter duration in cohort and fewer spirometry tests. Moreover, we did not find any associations between WS exposure and sputum methylation index (P = 0.90, Table 1), probably because moderate and heavy cigarette smoking is the driver for acquisition of sputum methylation in this smoker cohort.

### Effects of WS exposure on lung function decline

**Fig. 1:**
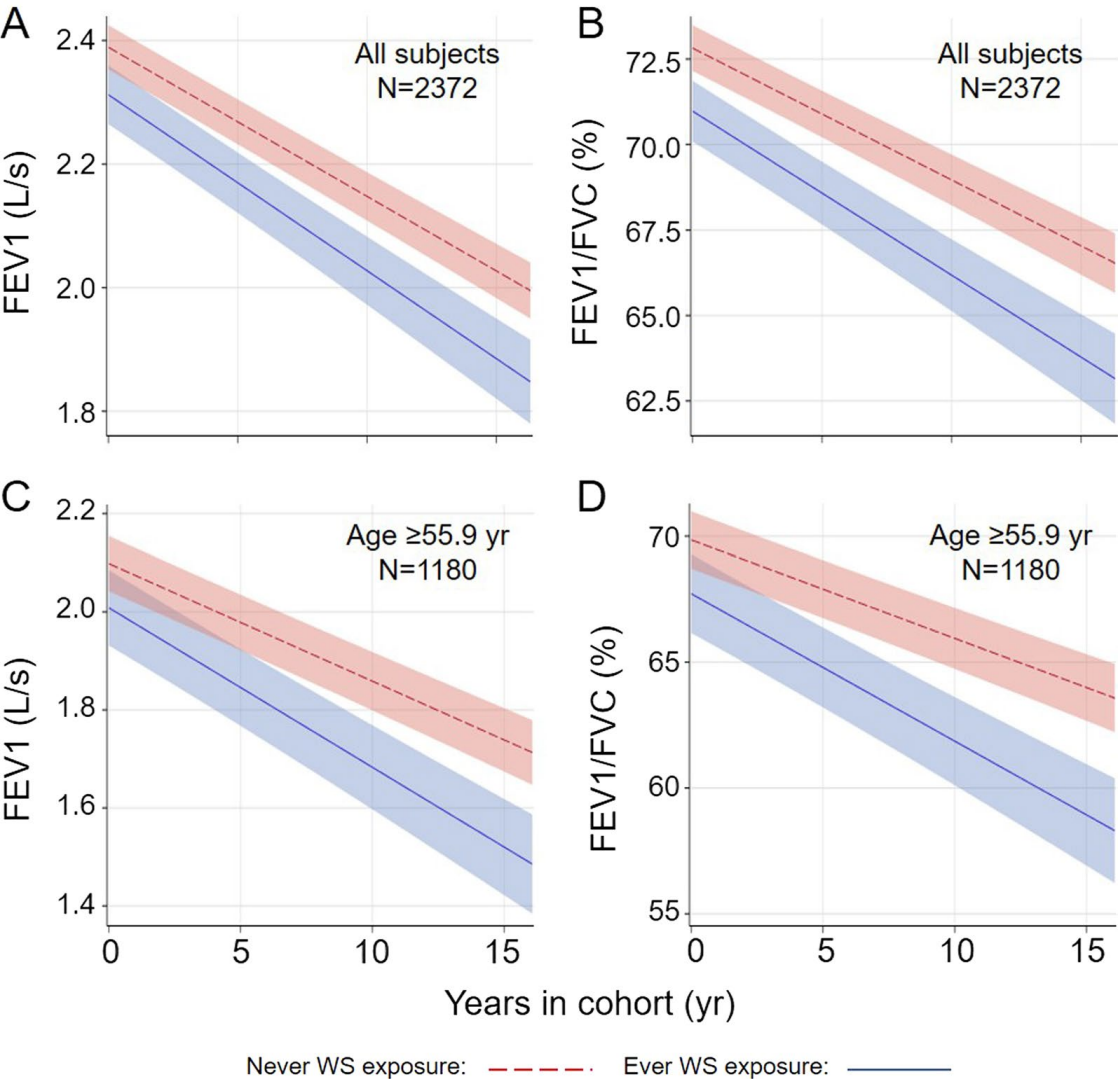
Wood smoke exposure accelerates decline of FEV1 and FEV1/FVC ratio in the Lovelace Smokers cohort. Subjects with “ever WS exposure for over a year” (n = 684) have a more rapid decline of FEV1 (by − 4.3 ml/s per year, P = 0.025, **A** and FEV1/FVC ratio (by − 0.093% per year, P = 0.015, **B** compared to those without (n = 1688). Stratification analysis by median age (55.9 year) identified a more robust impact of WS exposure on the decline of FEV1 (**C**) and FEV1/FVC ratio (**D**) in older smokers with the magnitude of effects doubling that seen in overall population. Average FEV1 and FEV1/FVC ratio over time in all subjects with and without WS exposure were plotted in non-Hispanic white females who smoked at baseline and had baseline age of 56.83 year, BMI of 28.27, height of 65.26 inch, and pack-years of 39.22. Average FEV1 and FEV1/FVC ratio over time in subjects ≥ 55.9 years old with and without WS exposure were plotted in non-Hispanic white females who smoked at baseline and had baseline age of 64.2 year, BMI of 28.14, height of 65.01 inch, and pack-years of 43.81

WS exposure was associated with lower FEV1 (− 75.2 ml/s, P = 0.0016) and FEV1/FVC ratio (-1.792%, P < 0.0001) at baseline, but had no impact on baseline FVC (Table 2). A significant impact of WS exposure on annual decline of FEV1 and FEV1/FVC ratio was also identified, but not for the decline of FVC (Table 2; Fig. 1A, B). WS exposure at baseline was associated with steeper slopes indicative of more rapid decline for FEV1 and FEV1/FVC ratio by 4.3 ml/s (P = 0.025) and 0.093% (P = 0.015), respectively. The defense mechanisms against environmental insults compromises as people age. Three-way interaction among WS exposure (yes versus no), baseline age (by year), and TIC (by year) was assessed using LME models and identified significant interactions for FEV1 (estimate = − 8.0 ml/s, P = 0.038) and FEV1/FVC ratio (estimate = − 0.184%, P = 0.017), but not for FVC (estimate = − 2.4 ml/s, P = 0.61). Stratification analyses by the median age of 55.9 year at study entry identified significant impact of WS exposure on the decline of FEV1 and FEV1/FVC ratio in older subjects (age ≥ 55.9 year, Table 2; Fig. 1C, D) but not in the younger group. However, WS exposure affected baseline FEV1 and FEV/FVC ratio regardless of age groups.

**Table 2:** Impact of ever WS exposure on baseline lung function and its decline^a^

Group	Years	Ever WS exposure	Ever WS exposure^a^ years
All subjects			
FEV1 (ml/s)	− 24.2 (1.0)	− 75.2 (23.7)	− 4.3 (1.9)
P value	< 0.0001	0.0016	0.025
FVC (ml)	− 15.0 (1.2)	− 26.1 (25.2)	− 2.4 (2.4)
P value	< 0.0001	0.30	0.30
FEV1/FVC ratio (%)	− 0.386 (0.019)	− 1.792 (0.430)	− 0.093 (0.038)
P value	< 0.0001	< 0.0001	0.015
Age < 55.9 year^b^			
FEV1 (ml/s)	− 24.4 (1.5)	− 65.6 (30.7)	− 0.5 (2.8)
P value	< 0.0001	0.033	0.85
FVC (ml)	− 13.5 (1.7)	− 7.3 (33.1)	− 1.6 (3.2)
P value	< 0.0001	0.82	0.62
FEV1/FVC ratio (%)	− 0.379 (0.029)	− 1.666 (0.493)	− 0.014 (0.053)
P value	< 0.0001	0.0008	0.80
Age ≥ 55.9 year^b^			
FEV1 (ml/s)	− 24.0 (1.2)	− 76.6 (36.8)	− 8.7 (2.6)
P value	< 0.0001	0.038	0.001
FVC (ml)	− 16.0 (1.6)	− 37.5 (38.7)	− 3.8 (3.5)
P value	< 0.0001	0.33	0.28
FEV1/FVC ratio (%)	− 0.391(0.026)	− 2.013 (0.724)	− 0.196 (0.055)
P value	< 0.0001	0.0055	0.0004

*WS* woodsmoke

^a^Linear mixed effects model was used to assess the impact of ever WS exposure on lung function decline through including an interaction term between ever WS exposure and time in cohort. We included fixed effects for baseline age, BMI, height, smoking status, and packyears, sex, and ethnicities, and random effects for intercept and time in cohort.

^b^Cohort was stratified based on a median age of 55.9 year

### Effects of WS exposure on lung function decline not confounded by smoking status, airway obstruction, and CMH

Our previous analyses identified associations of WS exposure with airway obstruction and CMH at baseline in 1861 LSC subjects enrolled prior to 2008 [13]. We replicated these analyses in a larger sample size in this study (n = 2372, Table 1). Because airway obstruction and CMH at baseline may increase the predisposition for lung function decline [40] and there was a weak correlation between WS exposure and baseline current smoking (P = 0.015, Table 1), we further analyzed whether the associations between WS exposure and decline of FEV1 and FEV1/FVC ratio were independent of baseline current smoking, airway obstruction, and CMH. Our analyses did find baseline airway obstruction and CMH were associated with a more rapid decline of FEV1 and FEV1/FVC ratio (Table 3). Inclusion of these three factors as main effects and their interactions with TIC individually or in combination only slightly (< 15%) reduced the magnitude of impact of WS exposure on lung function decline (Table 3). We further added baseline FEV1 or FEV1/FVC ratio as continuous variables and their interactions with TIC in the linear mixed effect models. Addition of these variables and their interaction terms with TIC did not reduce the magnitude of associations between woodsmoke exposure and lung function decline (data not shown).

**Table 3:** Impact of ever WS exposure on lung function decline independent of current smoking, airway obstruction, and CMH status at baseline^a^

Model	FEV1 (ml/s)	FEV1/FVC ratio (%)
Basic model		
WS exposure × years	− 4.3 (1.9)	− 0.094 (0.038)
P value	0.024	0.015
Basic model + current smoker × years		
WS exposure × years	− 4.1 (1.9)	− 0.088 (0.038)
P value	0.033	0.021
Current smoker × years	− 4.4 (1.7)	− 0.101 (0.033)
P value	0.0082	0.0026
Basic model + airway obstruction × years		
WS exposure × years	− 4.3 (1.9)	− 0.094 (0.039)
P value	0.025	0.016
Airway obstruction × years	− 2.9 (1.9)	− 0.113 (0.039)
P value	0.14	0.0041
Basic model + CMH × years		
WS exposure × years	− 4.0 (1.9)	− 0.088 (0.038)
P value	0.038	0.022
CMH × years	− 5.4 (2.0)	− 0.097 (0.040)
P value	0.0061	0.014
Basic model + current smoker × years + airway obstruction × years + CMH × years		
WS exposure × years	− 3.7 (1.9)	− 0.080 (0.037)
P value	0.050	0.034
Current smoker × years	− 3.9 (1.9)	− 0.102 (0.034)
P value	0.023	0.0026
Airway obstruction × years	− 7.2 (2.0)	− 0.279 (0.038)
P value	0.0002	< 0.0001
CMH × years	− 3.1 (2.1)	− 0.024 (0.041)
P value	0.13	0.56

*CMH* chronic mucous hypersecretion, *WS* woodsmoke

^a^Linear mixed effects model was used to assess the impact of ever WS exposure on lung function decline through including an interaction term between ever WS exposure and time in cohort (years). We included fixed effects for baseline age, BMI, height, smoking stats, and packyears, sex, and ethnicities, and random effects for intercept and time in cohort. Interactions terms for current smoking, airway obstruction, and CMH at baseline with time in cohort were added to test the independent components of ever WS exposure effects on decline of FEV1 and FEV1/FVC ratio. WS exposure, airway obstruction, and CMH were coded as binary variables. Time in cohort has year as the unit

### Multi-dimensional impact of WS exposure on health

WS exposure also had a strong impact on SGRQ and SF-36 scores and subscores even after adjusting for current smoking, comorbidity, airway obstruction, and CMH at baseline (Table 4). The difference of SGRQ scores and subscores between subjects with and without WS exposure all exceeded or approached the moderately clinically important difference for this instrument (≥ 8, [41, 42]). No interactions were identified between WS exposure and airway obstruction at baseline for affecting SGRQ scores (all Ps > 0.44), suggesting homogeneity of WS exposure effects across airway obstruction status. Promoter methylation of a 12-gene panel in sputum originally optimized for LC risk stratification in smokers by our group was recently shown to be capable of quantifying airway remodeling and predict lung function decline and all-cause mortality [37, 38]. Thus, to discover determinants for SGRQ scores, we further assessed whether sputum methylation affected SGRQ scores. Subjects with ≥ 3 genes methylated had higher symptom scores compared to subjects with 0–2 genes methylated (Additional file 1: Table S1). Additional adjustment of comorbidity, airway obstruction, and CMH at baseline in the model reduced the difference of symptom score by 40%, however the difference remained statistically significant. Individual gene methylation association analyses identified SULF2 and GATA4 as having the strongest associations with higher symptom scores if methylated. No significant associations were identified for activity, impact, or total scores.

**Table 4:** Impact of ever WS exposure on SGRQ and SF-36 scores independent of current smoking, comorbidity, airway obstruction, and CMH status at baseline

Score	Basic model^a^		Alternative model^b^	
	Estimate (SE)	P	Estimate (SE)	P
SGRQ				
Symptom	8.5 (0.9)	< 0.0001	5.7 (0.8)	< 0.0001
Activity	8.1 (1.0)	< 0.0001	5.4 (0.9)	< 0.0001
Impact	5.0 (0.6)	< 0.0001	3.3 (0.5)	< 0.0001
Total	6.9 (0.7)	< 0.0001	4.6 (0.6)	< 0.0001
SF-36				
Physical functioning	− 7.0 (1.1)	< 0.0001	− 4.6 (1.0)	< 0.0001
Role physical	− 11.0 (1.6)	< 0.0001	− 8.1 (1.6)	< 0.0001
Bodily pain	− 6.9 (1.1)	< 0.0001	− 5.6 (1.1)	< 0.0001
Role emotional	− 6.2 (1.8)	0.0005	− 4.0 (1.8)	0.023
Social functioning	− 5.6 (1.1)	< 0.0001	− 3.9 (1.1)	0.0004
Mental health	− 3.8 (0.9)	< 0.0001	− 2.9 (0.9)	0.0009
Vitality	− 5.9 (1.0)	< 0.0001	− 4.1 (1.0)	< 0.0001
General health perceptions	− 6.1 (0.9)	< 0.0001	− 3.8 (0.9)	< 0.0001

*SF-36* the short form 36 health survey questionnaire, *SGRQ* St. George’s Respiratory questionnaire, *WS* woodsmoke

^a^Basic model assessed the impact of ever WS exposure on SGRQ scores using linear mixed effects model or on SF-36 scores using generalized linear model

^b^Alternative model added Charlson comorbidity score (≥ 1 versus 0), airway obstruction, and CMH at baseline into the basic model to assess the independent components of effects for ever WS exposure

### Time to event analyses for lung cancer incidence and death

**Fig. 2:**
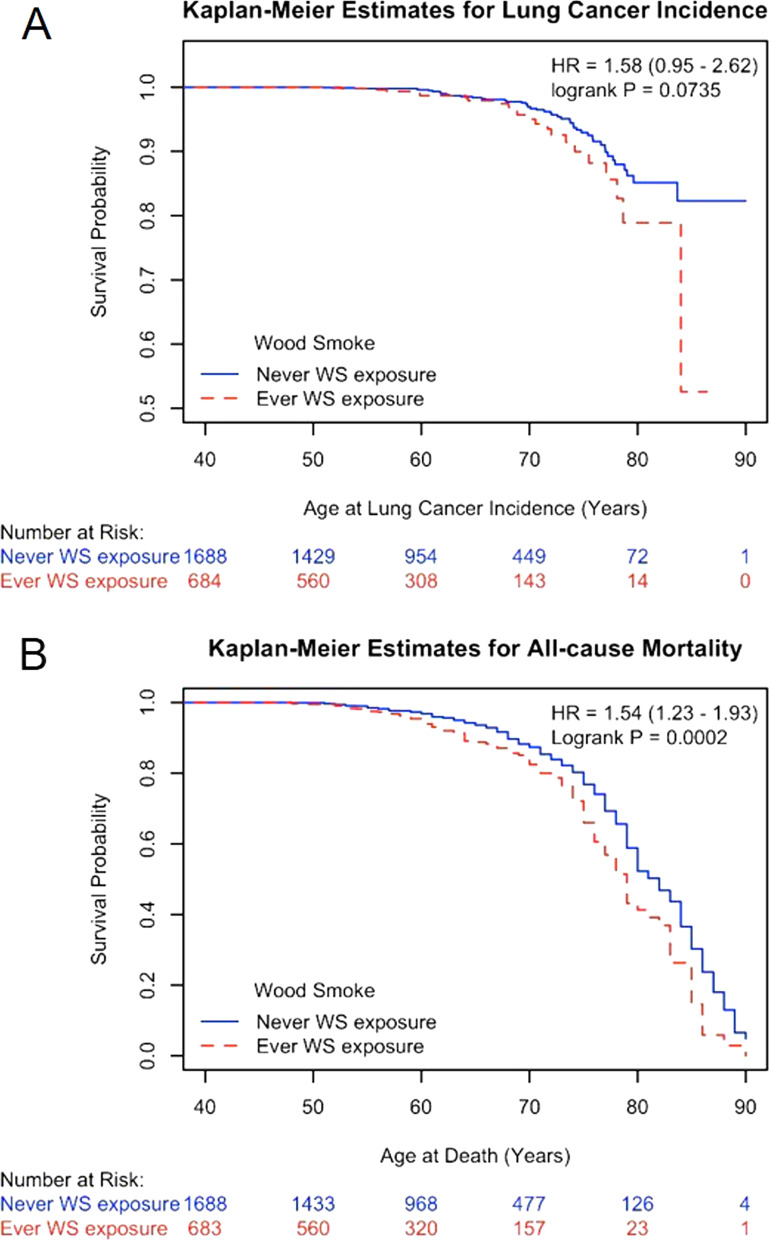
Kaplan–Meier curve for lung cancer incidence (**A**) and all-cause mortality (**B**) by wood smoke exposure in the Lovelace Smokers cohort. Till the middle of 2020, a total of 72 lung cancer incidences and 380 deaths were ascertained from 2372 LSC subjects during the follow-up period. Wood smoke exposure was associated with over 50% increased risk for lung cancer (unadjusted HR = 1.58, P = 0.0735, **A**) and all-cause mortality (unadjusted HR = 1.54, P = 0.0002, **B**). Age at death was missing for one subject with ever WS exposure

Cohort follow-up identified 72 LC incidences and 380 deaths through the middle of 2020. Time to event was calculated using date at birth (Table 5) or date at baseline visit (Additional file 1: Table S2) because the timing of ever WS exposure was unknown. Subjects with WS exposure had shorter time to event for LC incidence or death from all-causes or specific diseases, although the difference for LC incidence is of borderline (Fig. 2, data not shown). Subjects reporting WS exposure had a shortened lifespan by 3.5 year at death (Table 1). Cox regression analyses identified increased hazard ratio for lung cancer incidence or all-cause or disease-specific death, although the association for LC incidence is of borderline (Table 5, Supplemental Table 2). After the additional adjustment of baseline airway obstruction and comorbidity, the HR of all-cause mortality due to WS exposure remained significantly elevated (HR = 1.39, 95%CI = 1.10–1.75, P = 0.0054, not shown). These findings suggest that WS exposure increased hazard for LC incidence and all-cause and cardiopulmonary- and cancer-specific death by at least 50%.

**Table 5:** Ever WS exposure on lung cancer incidence and call-cause and disease-specific mortality

Endpoint	WS exposure	N	Event	Person-year^a^	HR (95%CI^b^	P
LC incidence	Yes	684	22	41,134	1.53 (0.92–2.55)	0.10
	No	1688	50	105,020		
All cause mortality	Yes	683	108	41,358	1.53 (1.21–1.92)	0.0003
	No	1688	272	105,980		
CPD mortality^c^	Yes	683	41	41,358	1.49 (1.03–2.15)	0.033
	No	1688	114	105,980		
Cancer mortality^d^	Yes	683	35	41,358	1.52 (1.02–2.28)	0.041
	No	1688	84	105,980		

*CPD* cardiopulmonary disease, *HR* hazard ratio, *LC* lung cancer, *WS* woodsmoke

^a^Person-year was calculated as age at last alive LC-free contact or age at LC diagnosis for lung cancer incidence analyses or age at last alive contact or age at death for mortality analyses. Age at death was missing for one subject with ever WS exposure

^b^Baseline values of age, smoking status, and packyears, sex, and ethnicity were included in Cox proportional hazards model for covariate adjustment for LC incidence. Education and income were included for additional covariate adjustment for mortality

^c^Deaths due to non-CPD causes were censored at the date of death

^d^Deaths due to non-cancer causes were censored at the date of death

### Health measurements mediating the association between WS exposure and all-cause mortality

We further analyzed whether the associations between WS exposure and all-cause mortality were mediated by health measurements (Table 6). This analysis included subjective (i.e., SGRQ and SF-36 scores) and objective (i.e., FEV1 and FEV1/FVC ratio) measurements that were significantly associated with all-cause mortality (all P values < 0.0003, not shown). Mediational effect sizes ranged from 4.1% to 21.1% with SGRQ total score and SF-36 physical functioning score mediating 21% of magnitude of association. This is comparable to if not higher than the mediational effect size seen for FEV1. Moreover, with additional adjustment for spirometry, significant associations between WS exposure and all-cause mortality remained.

**Table 6:** Mediational effects of health measurements on the associations between ever WS exposure and all-cause mortality

Potential mediator	Ever WS exposure^a^		Mediational effect size (%)^b^	Pperm^c^
	C (P = 0.0003)	C' (all Ps < 0.005)		
SGRQ score				
Symptom	0.42 (0.12)	0.37 (0.12)	12.0	0.03
Activity	0.42 (0.12)	0.35 (0.12)	17.0	< 0.005
Impact	0.42 (0.12)	0.35 (0.12)	17.9	< 0.005
Total	0.42 (0.12)	0.33 (0.12)	21.1	< 0.005
SF-36 score				
Physical functioning	0.42 (0.12)	0.33 (0.12)	20.9	< 0.005
Role physical	0.42 (0.12)	0.39 (0.12)	7.4	0.03
Role emotional	0.42 (0.12)	0.40 (0.12)	4.1	0.03
Social functioning	0.42 (0.12)	0.41 (0.12)	2.5	0.21
Mental health	0.42 (0.12)	0.39 (0.12)	6.8	0.02
Vitality	0.42 (0.12)	0.40 (0.12)	5.5	0.055
General health perceptions	0.42 (0.12)	0.36 (0.12)	15.5	< 0.005
Bodily pain	0.42 (0.12)	0.39 (0.12)	6.6	0.015
Spirometry				
FEV1	0.42 (0.12)	0.34 (0.12)	18.8	< 0.005
FEV1/FVC ratio	0.42 (0.12)	0.38 (0.12)	10.2	< 0.005

*WS* woodsmoke

^a^Cox proportional hazards model assessed the impact of WS exposure on all-cause mortality. Baseline values of age, smoking status, packyears, and annual income, education, sex, and ethnicity were included in Cox proportional hazards model for covariate adjustment. C was the estimate for WS exposure in model without individual potential mediators. C' was the estimate for WS exposure in model with individual potential mediators.

^b^Mediational effect size (%) was calculated as ([C–C'] × 100)/C

^c^Pperm was calculated using permutation based method. The relationship between survival data (survival time and censor status) and the vector of independent variables was permuted for 200 times. Each permutated database allowed the association analysis of all-cause mortality with ever WS exposure and other covariates without and with including individual potential mediators to calculate the C and C'. Permutation was conducted for 200 times to generate the distribution of C–C' under null hypothesis of no mediation. Value of C–C' calculated using observed data was compared to the distribution generated by permutation and Pperm was calculated as the number of permuted databases generating a C–C' that exceeded the observed value divided by 500

### Sensitivity analyses

Restricting analyses in subjects with 2 or more visits for spirometry or SGRQ did not change the results observed in the entire study cohort. Stratification analyses by baseline Charlson comorbidity score (≥ 1 versus 0) or education level (college or above versus high school or lower) were conducted to address potential issues of differential classification or confounding of social economic status, respectively (Additional file 1: Table S3). We used all-cause mortality as the outcome to ensure sufficient power in individual subgroup analyses. Significant associations were identified in subgroups with and without baseline comorbidities, suggesting the impact of WS exposure was highly unlikely due to differential misclassification. Moreover, significant associations were also identified in subgroups with and without college education or above, suggesting the impact of WS exposure was unlikely due to confounding from socioeconomic status. Among 380 cohort members who died, 96 subjects died within 4.86 years after study entry. So, additional sensitivity analyses were conducted by excluding these subsets. The estimates for FEV1 decline, FEV1/FVC ratio decline, and SGRQ total scores associated with WS exposure were − 4.3 (1.9) ml/year (P = 0.026), − 0.094 (0.038) %/year (P = 0.015), and 6.8 (0.7) (P < 0.0001) in the 2276 LSC subjects. Furthermore, excluding 72 LC incidents did not change the associations between WS exposure and lung function decline observed in the entire cohort. The estimates for FEV1 decline and FEV1/FVC ratio decline associated with WS exposure were − 4.6 (2.0) ml/year (P = 0.019) and − 0.097 (0.039) %/year (P = 0.012) in the 2300 LSC subjects.

## Discussion

Our study employed a combination of objective health measures (i.e., lung function decline, LC incidence, and disease mortality) and psychometric HRQoL assessment to delineate the pulmonary and overall health effects of WS exposure in a Southwestern USA cohort of middle-aged and older smokers. Compared to subjects without WS exposure, subjects with WS exposure had a more rapid decline of FEV1 (− 4.3 ml/s, P = 0.025) and FEV1/FVC ratio (− 0.093%, P = 0.015) but not of FVC (− 2.4 ml, P = 0.30). Age modified the impacts of WS exposure on lung function decline with stronger impacts seen in older subjects. Because decline in FVC was not associated with WS exposure, we propose that WS exposure leads to an obstructive pattern in COPD development [43]. It is important to note that the adverse effects of WS exposure on baseline FEV1 and FEV1/FVC ratio were independent of age at study entry. In addition to the explanation that WS exposure accelerates lung function decline, exposure to WS exposure at early life could also blunt lung development and impair the maximal attainable lung function, an established risk factor for development of COPD [44]. In addition, strong psychometric evidence was provided that WS exposure impaired all health domains assessed using the generic health SF-36 questionnaire and the lung disease-specific SGRQ. The effect of WS exposure on SGRQ scores doubled the minimal clinically important difference. For both categories of health measurements, we found significant components of the impacts of WS exposure independent of current smoking, airway obstruction, CMH, and comorbidity (for HRQoL measurements) at baseline. Finally, WS exposure was associated with lower age at death and cardiopulmonary diseases and cancers drove this association. Collectively, these findings provide strong support for an accelerated aging of the lung due to chronic WS exposure in a USA population with low exposure to ambient combustion emitted PM.

Carcinogenicity of WS exposure is supported by the abundance of human carcinogens (e.g., polycyclic aromatic hydrocarbons) detected in gaseous and particulate phases of WS and the mutagenicity and carcinogenicity of WS extract in both in vitro and preclinical models. However, carcinogenicity of ambient WS exposure has not been assessed as an independent agent in humans; instead it is considered as a component of outdoor air pollution and PM2.5, which were classified as group 1 carcinogen in humans by the International Agency for Research on Cancer. In 2006, indoor emissions from household combustion of biomass were classified as probably carcinogenic to humans (Group 2A) due to limited evidence for risk of lung cancer from epidemiologic studies. Several review and original research articles published after 2006 provided additional evidence supporting the associations between indoor biomass burning and risk for lung cancer and upper aero-digestive tract cancers mostly using cross-sectional case–control study design in both high- and low-income countries [25, 45–47]. Our study provides further evidence based on time-to-event analyses that WS exposure was associated with a > 50% increase risk for LC incidence and cancer-specific mortality, supporting the etiological role of WS exposure in carcinogenesis including LC.

WS composition varies by the type of biomass burned, burning conditions and stages, and secondary atmospheric reactions [48]. WS contains variable amounts of various toxic gases and particulate matters, with the latter containing carbonaceous cores covered by organic matters, metals, and inorganic salts. The majority of WS particles is in a nano-scale range and can deposit deep inside the lungs. Phagocytosis of WS particles by airway macrophages as a major clearance mechanism in acinar airway triggers persistent cytokine/chemokine secretion and generation of other mediators, such as exosomes for downstream toxicity. The development of COPD and lung cancer in susceptible people likely requires decades of repetitive exposure of airways to WS which may occur in the settings of household wood burning for winter heating and frequent and intense wild fires due to climate change in many regions (e.g., the Mountain West) in the United States. Similar to cigarette smoke or diesel exhaust, WS contains numerous established human carcinogens, e.g., polycyclic aromatic hydrocarbons. Moreover, WS contains much higher levels of carbonaceous cores than cigarette smoke [49, 50]. Our study of occupational exposure to black carbon identified a dose–response relationship between macrophage carbon load and genomic instability in peripheral blood, an established cancer biomarker which may be mediated by a mutagenic cytokine TNF-α in circulation [51]. Recent studies from Tesfaigzi’s group identified in vivo and in vitro evidence supporting WS-induced mucous cell hyperplasia which is more prominent in p53 arginine carriers than proline carriers [52]. Fractionation of WS extract identified oxalate that recapitulated the MUC5AC induction by whole WS extract in human primary airway epithelial cells with p53 arginine genotype [52]. These studies provide initial evidence that WS exposure and its specific constituent could induce mucous cell hyperplasia which underlies a specific sub-phenotype of COPD, i.e., chronic bronchitis. Findings of these studies begin to delineate how WS exposure interacts with host factors in COPD development. More researches are needed in this field to explore the molecular mechanisms underlying WS-induced COPD and lung cancer and the involvement of secondary reactions in determining toxicity and health effects of WS exposure.

Concomitant gene methylation detected in exfoliated lung epithelial cells collected in sputum provides an assessment of the extent of field cancerization and is a validated biomarker for diagnosis of primary LC and its recurrence [53, 54]. Moreover, an optimized 12-gene methylation panel in sputum was strongly associated with higher prevalence of CMH, a more rapid decline of FEV1, and increased all-cause mortality in the LSC and Pittsburgh Lung Screening Study cohort [37, 38]. Those 12 genes confer essential functions in biological pathways of cell cycle, cellular senescence, DNA repair, apoptosis, RAS signaling, and invasion that are mechanistically implicated in chronic lung injury and COPD development [14, 15, 55]. The current study identified a strong association between sputum methylation and self-rated SGRQ symptom score with the majority of effect independent of comorbidity and lung diseases, further supporting sputum methylation as an epigenetic endo-type biomarker for lung health [38, 56]. Although only baseline sputum data is available for analyses in this study, the longitudinal collection of sputum samples in the LSC offers a unique opportunity to assess the trajectory of sputum methylation over time and the role of this trajectory in lung aging.

Differential misclassification occurs when misclassification of exposure is not equal between subjects with or without certain health outcomes. Although subjects with WS exposure have higher Charlson comorbidity score at baseline, it is highly unlikely that our results were biased by differential misclassification. First, the LSC is a community-based volunteer cohort enrolling moderate and heavy smokers who have concerns about their health due to past and/or ongoing smoking history. Previous WS exposure should be something that they are least concerned about. Second, our stratification analyses by baseline comorbidity did not identify any difference in magnitude of associations between WS exposure and all-cause mortality, further supporting the very unlikelihood of differential misclassification. It is also unlikely that our results were confounded by lower socioeconomic status associated with WS exposure. Because baseline annual income has high missing rate (21%) an does not capture the wealth information well for the elderly in this cohort, we used education levels as the proxy for social economic status [57]. No difference was identified for the magnitude of associations of all-cause mortality with WS exposure between subjects with and without college education or above. Moreover, education levels and baseline income were included for adjustment in association analyses for mortality.

Although our question for assessing WS exposure presumably provides a qualitative readout of WS exposure for over a year from any exposure sources and has been initially validated by showing strong associations with lung function and pulmonary comorbidities [13], this binary exposure assessment does not allow us to detect dose–response relationship. Currently, we are developing an artificial intelligence based counting algorithm for macrophage carbon load assay which assesses the lung dose of combustion emitted particulate matter from all sources [58]. Periodical assessment of macrophage carbon load will provide a valid dosimetry over time and allows precise assessment of dose–response relationship.

## Conclusions

This study identified epidemiological evidence supporting WS exposure as an independent etiological factor for the development of COPD through accelerating lung function decline in an obstructive pattern. Our study also provides prospective analyses supporting the escalation of carcinogenicity for WS exposure. This study stresses the importance of further characterization of the dose–response relationship between WS exposure and health effects, a key component for risk assessment of residential WS exposure in the USA and other high-income countries. This field of research is very timely as millions of people are not only exposed to residential WS but also to ambient WS from yearly wildfire episodes that are becoming more frequent and prolonged [1].

## Supplementary Information

**Additional file 1: Table S1**. Impact of sputum methylation on SGRQ scores (n=1798). **Table S2**. Ever WS exposure on lung cancer incidence and call-cause and disease-specific mortality. **Table S3**. Stratification analyses of the impacts of WS exposure on all-cause mortality by baseline comorbidity or education levels.

## Data Availability

Data is available in de-identified form upon completion of Data Use Agreement with Lovelace Biomedical Research Institute who owns the data.

## Abbreviations

WS: Wood smoke
COPD: Chronic obstructive pulmonary disease
LC: Lung cancer
LSC: Lovelace smokers cohort
SGRQ: St. George’s respiratory questionnaire
