# Supplementary material for: Wood smoke exposure affects lung aging, quality of life, and all-cause mortality in New Mexican smokers
Source: Respir Res. 2022 Sep 8;23:236. doi: 10.1186/s12931-022-02162-y (PMC9454202; doi:10.1186/s12931-022-02162-y)
Supplement: Supplementary file 1 — Additional file 1: Table S1. Impact of sputum methylation on SGRQ scores (n=1798). Table S2. Ever WS exposure on lung cancer incidence and call-cause and disease-specific mortality. Table S3. Stratification analyses of the impacts of WS exposure on all-cause mortality by baseline comorbidity or education levels. [file 12931_2022_2162_MOESM1_ESM.docx]

Supplemental Table 1. Impact of sputum methylation on SGRQ scores (n=1798)

| Group comparison* | Score | Basic model† | |  | Alternative model‡ | |
| --- | --- | --- | --- | --- | --- | --- |
|  |  | Estimate (SE) | P |  | Estimate (SE) | P |
| MI, ≥3 versus 0-2, 39.4% | Symptom | 3.4 (0.9) | 0.0002 |  | 2.0 (0.8) | 0.012 |
| MI, ≥3 versus 0-2, 39.4% | Activity | 1.2 (1.1) | 0.25 |  |  |  |
| MI, ≥3 versus 0-2, 39.4% | Impact | 0.5 (0.6) | 0.45 |  |  |  |
| MI, ≥3 versus 0-2, 39.4% | Total | 1.2 (0.8) | 0.10 |  | 0.2 (0.7) | 0.72 |
| P16, 1 versus 0, 20.3% | Symptom | 0.6 (1.1) | 0.61 |  |  |  |
| MGMT, 1 versus 0, 25.9% | Symptom | 0.4 (1.0) | 0.69 |  |  |  |
| DAPK, 1 versus 0, 16.3% | Symptom | 1.2 (1.2) | 0.31 |  |  |  |
| RASSF1A, 1 versus 0, 0.7% | Symptom | 10.0 (5.7) | 0.079 |  | 6.4 (4.9) | 0.19 |
| GATA4, 1 versus 0, 36.3% | Symptom | 2.7 (0.9) | 0.004 |  | 1.8 (0.8) | 0.028 |
| GATA5, 1 versus 0, 14.6% | Symptom | 2.3 (1.3) | 0.066 |  | 1.8 (1.1) | 0.090 |
| PAX5α, 1 versus 0, 14.4% | Symptom | -1.4 (1.3) | 0.27 |  |  |  |
| PAX5β, 1 versus 0, 7.8% | Symptom | 0.8 (1.7) | 0.62 |  |  |  |
| DAL1, 1 versus 0, 7.3% | Symptom | 2.5 (1.7) | 0.15 |  |  |  |
| SULF2, 1 versus 0, 35.1% | Symptom | 3.7 (0.9) | <0.0001 |  | 2.2 (0.8) | 0.0056 |
| PCDH20, 1 versus 0, 36.0% | Symptom | 2.6 (0.9) | 0.0062 |  | 1.5 (0.8) | 0.057 |
| JPH3, 1 versus 0, 21.4% | Symptom | 1.0 (1.1) | 0.37 |  |  |  |

Definition of abbreviations: MI, methylation index; SGRQ, St. George’s Respiratory questionnaire

* Group comparison listed includes variable name, comparison, and percentage of cohort members with the change (e.g., ≥3 for MI or 1 for P16 methylation).

† Basic model assessed the impact of sputum methylation on SGRQ scores using linear mixed effects model. We included fixed effects for baseline age, BMI, height, smoking status, and packyears, sex, ethnicities, education, and ever WS exposure, and random effects for intercept and time in cohort.

‡ Alternative model added Charlson comorbidity score (≥1 versus 0), airway obstruction, and CMH at baseline into the basic model to assess the independent components of effects for sputum methylation.

Supplemental Table 2. Ever WS exposure on lung cancer incidence and call-cause and disease-specific mortality

| Endpoint | WS exposure | N | Event | Person-year | HR (95%CI) | P |
| --- | --- | --- | --- | --- | --- | --- |
| LC incidence | Yes | 684 | 22 | 3264 | 1.60 (0.95 – 2.68) | 0.076 |
|  | No | 1688 | 50 | 9256 |  |  |
| All cause mortality | Yes | 683 | 108 | 3556 | 1.55 (1.23 – 1.94) | 0.0002 |
|  | No | 1688 | 272 | 10217 |  |  |
| CPD mortality | Yes | 683 | 41 | 3556 | 1.54 (1.07 – 2.23) | 0.021 |
|  | No | 1688 | 114 | 10217 |  |  |
| Cancer mortality | Yes | 683 | 35 | 3556 | 1.55 (1.04 – 2.31) | 0.034 |
|  | No | 1688 | 84 | 10217 |  |  |

Definition of abbreviations: CPD, cardiopulmonary disease; HR, hazard ratio; LC, lung cancer; WS, woodsmoke

* Person-year was calculated as age at last alive LC-free contact or age at LC diagnosis minus age at baseline for lung cancer incidence analyses or age at last alive contact or age at death minus age at baseline for mortality analyses. Age at death was missing for one subject with ever WS exposure.

† Baseline values of age, smoking status, and packyears, sex, and ethnicity were included in Cox proportional hazards model for covariate adjustment for LC incidence. Education and income were included for additional covariate adjustment for mortality.

‡ Deaths due to non-CPD causes were censored at the date of death.

§ Deaths due to non-cancer causes were censored at the date of death.

Supplemental Table 3. Stratification analyses of the impacts of WS exposure on all-cause mortality by baseline comorbidity or education levels

| Variable | Subgroup | All-cause mortality* | |
| --- | --- | --- | --- |
|  |  | HR (95%CI) | P |
| Charlson comorbidity score |  |  |  |
|  | 0 | 1.67 (1.08 – 2.59) | 0.022 |
|  | ≥1 | 1.35 (1.03 – 1.78) | 0.032 |
| P for interaction† |  |  | 0.50 |
| Education |  |  |  |
|  | ≤12 years | 1.90 (1.24 – 2.91) | 0.003 |
|  | >12 years | 1.38 (1.04 – 1.83) | 0.026 |
| P for interaction† |  |  | 0.30 |

Definition of abbreviations: SGRQ, St. George’s Respiratory questionnaire; HR, hazard ratio

* Cox regression was used to assess the impact of WS exposure on all-cause mortality. Baseline values of age, smoking status, packyears, and income, sex, ethnicity, and education (for stratification analysis by comorbidity only) were included in the model for covariate adjustment.

† Interaction assessed whether the impact of WS exposure varied by stratification variables.
